# Signal Suppression in LC-ESI-MS/MS from Concomitant Medications and Its Impact on Quantitative Studies: An Example Using Metformin and Glyburide

**DOI:** 10.3390/molecules28020746

**Published:** 2023-01-11

**Authors:** Jingyu Liu, Fulin Jiang, Zihan Lu, Chang Zhang, Peiqing Liu, Min Huang, Guoping Zhong

**Affiliations:** 1 Guangdong Provincial Key Laboratory of New Drug Design and Evaluation, Institute of Clinical Pharmacology, School of Pharmaceutical Sciences, Sun Yat-sen University, Guangzhou 510080, China; 2 National and Local United Engineering Lab of Druggability and New Drugs Evaluation, School of Pharmaceutical Sciences, Sun Yat-sen University, Guangzhou 510080, China

**Keywords:** liquid chromatography-tandem mass spectrometry, concomitant medication, signal suppression, quantitative analysis

## Abstract

Liquid chromatography-tandem mass spectrometry (LC-MS/MS) has been widely used in the quantitative analysis of drugs. The ubiquitous concomitant drug scenario in the clinic has spawned a large number of co-analyses based on this technique. However, signal suppression caused by concomitant drugs during electrospray ionization may affect the quantification accuracy of analytes, which has not received enough attention. In this study, metformin (MET) and glyburide (GLY) were co-eluted by the conventional optimization of chromatographic conditions to illustrate the effect of signal suppression caused by the combined drugs on the quantitative analysis. The response of MET was not affected by GLY over the investigated concentration range. However, the GLY signal could be suppressed by about 30% in the presence of MET, affecting its pharmacokinetic analysis in simulated samples. As an attempt to solve the suppression of GLY by co-eluting MET, dilution can alleviate the suppression. However, this method still has limitations due to the sacrifice of sensitivity. The stable isotope-labeled internal standard could play a role in correction and improve the quantitative accuracy of GLY, which was further confirmed in the pharmacokinetic study of simulated samples. This study provided an example model to illustrate the possible effect of clinical drug combination on LC-MS/MS drug quantitative analysis and investigated the effective methods to solve this problem.

## 1. Introduction

The liquid chromatography-tandem mass spectrometry (LC-MS/MS) technique combines the high separation ability of liquid chromatography with the high selectivity and sensitivity of mass spectrometry, and it has wide application in the quantitative detection of drug concentration in biological samples. At present, simultaneous quantification of multiple analytes using LC-MS/MS is very common. Mass spectrometry can distinguish the target analyte from other substances by monitoring selected mass ions. This advantage allows the analytes to be detected at the same time, which greatly reduces the analysis time [1].

Although the mass spectrum signal has extremely high specificity without worrying about the influence of co-analytes, the signal strength is at risk of being affected. The main reason is that the ionization process of analytes in the ion source is easily affected by co-eluents, which shows ion suppression or enhancement (matrix effect). The matrix effect was initially thought to be caused by endogenous substances in the matrix of the biological samples, but it was later found that co-elution of drugs, internal standards, and metabolites could all suppress/enhance analytes response [2,3,4,5]. Based on several models of electrospray ionization (ESI) [6], the mechanism of matrix effect has also been further explored [7,8]. The possible mechanisms of ion suppression are charge competition and the change of surface tension of charged droplets, while the mechanism of ion enhancement is not clear [9,10,11]. For the ion interference caused by the endogenous matrix in biological samples, many studies have reported a variety of elimination or correction methods. The optimization of pretreatment methods and chromatographic separation is considered to be effective measures to reduce matrix effect, and the correction effect of stable isotope-labeled internal standard (SIL-IS) on matrix effect is also widely discussed and applied [12]. In addition, dilution can also reduce the degree of matrix effect [13,14]. However, there is still a lack of systematic research on the applicability of the above methods to solve the ion interference from co-eluting drugs.

In method validation, matrix effect is usually evaluated to avoid the impact on quantitative analysis [15,16]. However, the composition of the blank biological matrix used in method validation may lack the co-elution of drugs, or be at different concentrations than the actual biological samples, so the results of validation could not reflect the interference to the analyte signal from concomitant medications in the clinical samples. The European Bioanalysis Forum (EBF) has considered the interference of co-medication on bioanalysis [17]. LC-MS/MS has higher selectivity than ligand binding assay and can prevent the interference of combined drugs. In addition to this interference due to the low selectivity, ion suppression or enhancement caused by co-eluting drugs in LC-MS/MS is also noteworthy. For the improvement of method validation, it has been proposed to inject co-eluting drugs possibly present in biological samples together with the analytes into the mass spectrometry to explore the existence of interference [18]. Nevertheless, the complexity of clinical drug combination may make it difficult for analysts to fully grasp the possible drugs. The drug combination is common, and patients’ medication history is often incompletely recorded, with 61% of patients having one or more unregistered drugs in hospital medical records [19]. Signal suppression by co-elution would be difficult to be aware of if a combined drug is not registered in the drug history and co-eluted with the analytes in bioanalysis. In addition, when multiple substances are detected at the same time, the combined drugs are particularly prone to co-elution in a short analysis time, which may double the validation workload. It is undeniable that the use of standards for validation is a strategy for the identified concomitant medication that can produce interference. For the unpredictable drug combination, it is necessary to find a more reasonable and reliable correction strategy. Given the excellent performance of SIL-IS in correcting the matrix effect, this study further confirmed its effectiveness in correcting ion interference caused by co-eluting drugs.

Metformin (MET) and glyburide (GLY) as antidiabetic agents with different mechanisms are commonly used in combination for the treatment of type 2 diabetes. The combination drug containing glyburide and metformin hydrochloride as active ingredients (tablets), approved by the Food and Drug Administration (FDA) in 2004, is still in clinical use [20]. MET and GLY are usually determined individually or simultaneously to investigate the pharmacokinetics, evaluate the bioequivalence of commercially available tablet formulations, or optimize the dose administered in combination therapy [21]. Therefore, MET and GLY were applied as the model drugs in this study to evaluate the impact of the signal interference caused by the co-eluting combined drugs on quantitative analysis and pharmacokinetics, and then to confirm the applicability of solutions including chromatographic separation, dilution, and SIL-IS correction.

## 2. Results and Discussion

### 2.1. The Co-Eluting Chromatographic Method for Metformin and Glyburide

**Figure 1:**
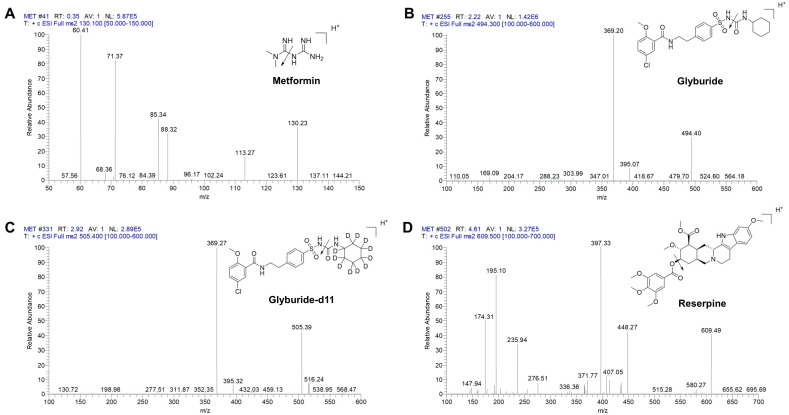
Two–stage full–scan mass spectrum of the analytes and internal standards. (**A**) Metformin, (**B**) Glyburide, (**C**) Glyburide-d11, (**D**) Reserpine.

**Figure 2:**
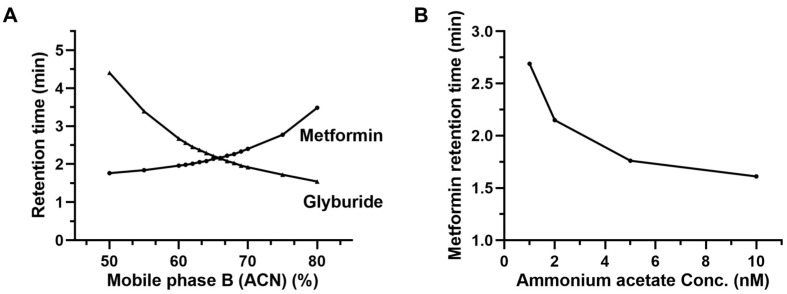
Changes in retention time of analytes with mobile phase composition. (**A**) Change of retention behavior of analytes with a mobile phase composed of acetonitrile (ACN)-water containing 2 mM ammonium acetate. (**B**) Change of retention behavior of MET with different concentrations of ammonium acetate (the concentration ratio of acetic acid to ammonium acetate was constant) at 65% organic phase.

**Figure 3:**
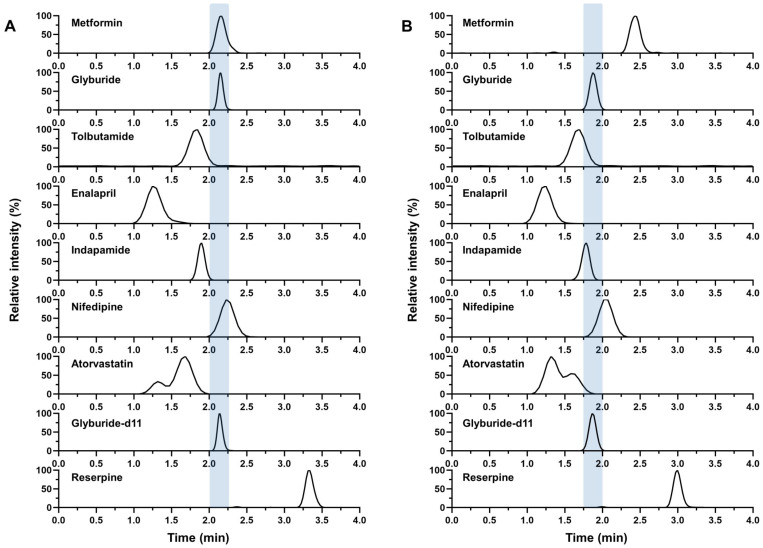
Typical chromatograms of the MET, GLY, internal standards and other concomitant medications. (**A**) MET and GLY chromatographic peaks overlapped under the condition of acetonitrile–water containing 2 mM ammonium acetate (65:35, *v/v*), (**B**) MET and GLY chromatographic peaks were separated under the condition of acetonitrile–water containing 2 mM ammonium acetate (71:29, *v/v*). MET: metformin; GLY: glyburide.

Chromatographic conditions are critical for the establishment of co-analytical models and subsequent analysis. The structure of MET and GLY is given in Figure 1, and the polarity difference between MET and GLY is obvious (Log *p*, −2.31 and 3.75). Hence, ammonium acetate and acetic acid were added to the aqueous phase to adjust the retention behavior of MET and GLY according to the reported mobile phase composition [22]. Ammonium acetate was found to have a signal suppression effect on GLY, but its ability to regulate MET retention time was superior to formic acid and water, and the chromatographic peak shape of MET was also improved. Therefore, the ammonium acetate concentration was determined to be 2 mM. Peracids can cause poor retention of MET, while neutrality can lead to peak tailing. So, the pH of the aqueous phase adjusted with acetic acid was 5.3 (±0.1). Under the chromatographic conditions in this study, the influence of mobile phase composition on the retention behavior of MET and GLY is shown in Figure 2. The retention time of MET in reverse-phase chromatography increased with the increase in the organic solvent composition of the mobile phase. We hypothesized that the binding force between the weakly acidic silanol group and the basic MET enhanced the retention ability of MET in the column and that the amount of ammonium acetate and acetic acid affected the magnitude of the force. Ammonium acetate competitively bound to the silanol group and acetic acid reduced the pH of the mobile phase to reduce the number of free silanol groups. Therefore, with the increase in the organic phase ratio, the decrease in ammonium acetate and acetic acid led to the enhanced binding of the silanol group to MET, resulting in increased retention time of MET in the column. To support the guess, the effect of different concentrations of ammonium acetate and acetic acid on MET retention at a constant organic phase ratio was investigated. According to the concentration commonly used in the laboratory, the aqueous phase containing 1, 2, 5, and 10 mM ammonium acetate (the concentration ratio of acetic acid to ammonium acetate was constant) was prepared, and the retention time of MET was investigated at 65% organic phase. The results are shown in Figure 2B. It can be seen that the retention time of MET increased nonlinearly with the decrease in the concentration of ammonium acetate and acetic acid. This supports the result that when the organic phase ratio was increased, the concentrations of ammonium acetate and acetic acid were decreased, and the retention time of MET was increased. This further supports the hypothesis that a decrease in the concentration of ammonium acetate and acetic acid weakened the shielding effect on the silanol groups, resulting in the enhanced binding of the silanol group to MET in the column and an increase in MET retention time. To explore the suppression of the two co-eluting analytes, the ratio of mobile phase A to B was determined to be 35:65 (*v/v*), and the retention time for both was 2.16 min. Typical chromatograms of the analytes are shown in Figure 3A. It is worth mentioning that the overlapping of chromatographic peaks of MET and GLY in this LC method is exactly the best scheme to avoid the retention time of both approaches to the dead time (about 1.4 min) to reduce the interference of the biological matrix. However, the following is the signal interference problem that may be caused by the co-elution of the two.

### 2.2. Signal Suppression between Metformin and Glyburide

In this study, the signal suppression of MET and GLY was first investigated using solutions at five concentration levels. The use of samples at the five concentrations allowed for the investigation of signal suppression over the entire calibration range. Signal suppression for the two analytes at five concentration levels is shown in Table 1. The occurrence of signal suppression was determined by comparing the response of the analyte in a mixed sample containing both MET and GLY with that of the analyte in a sample containing only the analyte. Less than 85% signal change indicates signal suppression. The results showed that the mean rate of signal change for MET all met the range of 85–115% [15], indicating that it was not affected by co-elution GLY over the concentration range investigated. However, GLY signals were significantly suppressed by high concentrations of MET, with a maximum suppression rate of 66%, which may affect the accurate quantification of GLY. From the results in Table 1, it seems that the degree of signal suppression of GLY was not significantly related to the concentration of GLY, but increased with the concentration of MET. This phenomenon indicated that the extent of the signal suppression of GLY by MET did not depend on the analyte concentration, but was influenced by the matrix concentration, that is, the concentration of the interfering substance, which was similar to the phenomenon in the matrix effect study by Stahnke et al. [14]. In addition, MET and GLY were separated by chromatography to further confirm that the signal change of GLY is caused by MET co-eluting. To separate MET and GLY chromatographically, the mobile phase B (acetonitrile, ACN) ratio was increased to 71% (Figure 3B), and the experiment in this section was repeated under this chromatographic condition. The results showed that the mean rate of signal change for MET and GLY all met the range of 85–115%; in other words, there was no signal suppression between MET and GLY.

To simultaneously extract MET and GLY from plasma samples and minimize the influence of matrix effects, tert-butyl methyl ether (MTBE) coupled ACN were selected for sample treatment at volumes of MTBE and ACN, 300 μL and 150 μL, respectively, after exploring their influence on recovery and matrix effects (Figure S1, Supplementary Materials). Under the final sample pretreatment conditions, the recovery and matrix effect are shown in Table S1 (Supplementary Materials). After pretreatment of plasma samples, the recoveries of MET and GLY were about 25% and 62%, respectively, and no significant matrix effect was observed. Therefore, their actual concentrations have decreased, but the GLY signal was still significantly suppressed by MET (Table 1). This represents a possible situation in sample analysis. Combined drugs still coexist after routine pretreatment and co-elute after the chromatographic process, resulting in ionization interference.

Although the exact mechanism of the matrix effect has not been elucidated, signal suppression between co-eluting drugs can be explained by the equilibrium model developed by Enke [23]. The solution containing analytes forms small charged droplets through a high voltage electric field, then undergoes fission and solvent evaporation to transform into gas phase ions and enter the mass analyzer. In the process, substance ions such as co-eluting drugs or endogenous compounds in the matrix and analytes ions all compete to supply a fixed number of surface charges, resulting in signal suppression. Moreover, due to the different structures between the co-analytes, the mutual ion interference between them is likely to show different degrees of signal suppression. The compounds that have the higher proton affinity should be more easily ionized [24]. The characteristic guanidine group of MET gives it a higher proton affinity. Therefore, the signal suppression of GLY by MET might be due to the competition of MET for the excess charge on the surface of charged droplets. The GLY charge may be lost through neutralization reactions or charge transfer due to the presence of MET with higher proton affinity, resulting in the decrease in the GLY signal [24,25]. Although the concentration of MET in plasma samples decreased relatively because of the low recovery, MET still suppressed the GLY signal to some extent. In short, the mechanism of signal suppression from co-eluting drugs is similar to that from the biological matrix, and they both can pose potential risks to accurate quantitative analysis.

### 2.3. Strategies to Solve Signal Suppression of Glyburide by Co-Eluting Metformin

#### 2.3.1. Chromatographic Separation

The LC-MS/MS detection method provides convenience for the simultaneous detection of multiple components. Drug co-eluting during the simultaneous analysis of multiple concomitant medications in a short analysis time is unavoidable. Under the condition of chromatographic overlap and separation of GLY and MET, the chromatographic retention behaviors of several antidiabetic drugs, antihypertensive drugs and lipid-lowering drugs were investigated simultaneously. Figure 3 shows that even if chromatographic separation of MET and GLY was achieved, GLY still overlapped with other drugs. This phenomenon suggests that the chromatographic separation method is not a foolproof solution. After the two analytes are chromatographic separated, the analyte may also co-elute with other concomitant medicine and generate signal suppression/enhancement. Moreover, chromatographic separation usually prolongs analysis time and increases the difficulty of method establishment. The chromatographic separation of combined drugs needs to comprehensively consider the optimization of the elution gradient, the composition, the pH of the mobile phase, the adjustment of flow rate, the applicability of the chromatographic column, etc. [23]. In addition, some drug combinations containing structurally similar drugs may have problems achieving chromatographic separation. Therefore, theoretically, chromatographic separation can solve the ion suppression of co-eluting substances, but there are many limitations in practical application. So, it is necessary to find a more effective solution to signal suppression under the condition of co-elution.

#### 2.3.2. Sample Dilution

From the results in Table 1, it is speculated that the degree of suppression of the GLY signal may be related to the concentration of MET. Therefore, it is assumed that dilution reduces the concentration of MET in the sample to mitigate the signal suppression effect of MET on GLY. Then, the lower limit of quantification (LLOQ), low-quality control (LQC), medium-quality control (MQC), high-quality control (HQC), and upper limit of quantitation (ULOQ) samples of MET and GLY were diluted 10- or 20-fold. The results of the signal suppression between MET and GLY after dilution are also shown in Table 1. The results show that after 10- or 20-fold dilutions, the suppression of GLY by MET decreased, but MET could still suppress the GLY signal at HQC and ULOQ levels. Moreover, the relative standard deviation (RSD) of several GLY samples at LLOQ and LQC concentration levels exceeded 20% at a 20-fold dilution. The main reason was that the response of GLY dropped to close to the quantitative limit of the signal-to-noise ratio of 10, and there were background disturbances such as baseline disturbance. Therefore, dilution can play a role in reducing the degree of ion suppression, but it may not be able to reliably solve the signal suppression between co-eluting substances. The increase in dilution multiple can also affect the sensitivity of the quantitative method, resulting in limitations.

#### 2.3.3. Correction of Stable-Isotope-Labeled Internal Standard

**Figure 4:**
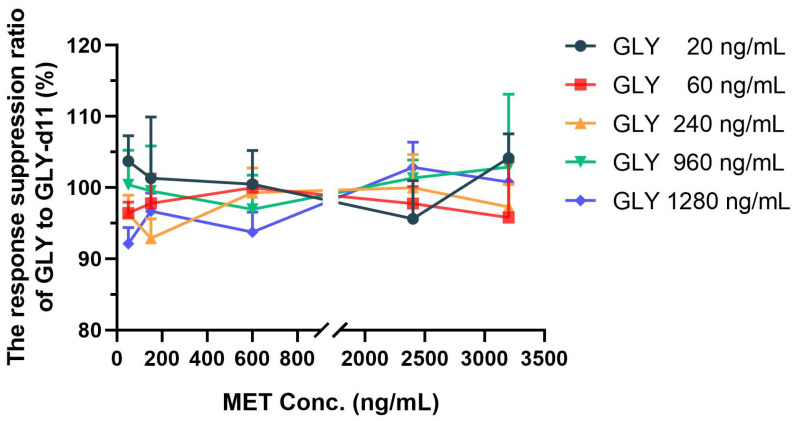
The response suppression ratio of GLY to GLY-d11 at different concentrations of GLY and MET (*n* = 3). MET: metformin; GLY: glyburide; GLY-d11: glyburide-d11.

Stable-isotope-labeled analogs and the corresponding analytes have similar structure and physical and chemical properties, so SIL-IS is often used to correct the matrix effect of the analyte. In this study, the SIL-IS working solution was added and the degree of signal suppression of GLY and glyburide-d11 (GLY-d11) by MET were compared. The results in Figure 4 show that the ratio of signal suppression of MET on GLY to that on GLY-d11 was close to 100%, that is to say, GLY-d11 was equally suppressed by MET as GLY, and finally the ratio of analytes to SIL-IS was not affected by signal suppression. According to the results, it is speculated that GLY-d11 may improve the accurate quantification of GLY affected by signal suppression. It should be noted that the SIL-IS method also has some limitations. Studies have shown that signal suppression can also occur between the analytes and their SIL-IS [2,3,4,5]. It has been suggested that the ratio of the analyte to the internal standard is proportional to the concentration of the analyte, without affecting the accuracy of the quantification of the analyte, even though the signal suppression may affect the limit of detection [26]. However, Liang et al. [4] found that this mutual suppression would affect the assay reproducibility, accuracy, and linearity besides sensitivity if an inappropriate SIL-IS concentration was selected. In the simultaneous detection of multiple substances, the SIL-IS may be used not only as the internal standard of the unlabeled analog, but also of other analytes. In this case, when the SIL-IS is affected by the co-eluting unlabeled analog, it will also affect the quantitative analysis of other analytes or metabolites [2,3]. If mutual suppression/enhancement between the SIL-IS and the analyte is detected, the impact of suppression on quantitative analysis should be carefully evaluated. In addition, the use of deuterium in place of hydrogen in the deuterated SIL-IS slightly alters the lipophilicity of the molecule, thereby altering the retention of the deuterated SIL-IS in the column. The slight retention time difference between the deuterated SIL-IS and the analyte may result in different signal interference degrees, resulting in a change in the ratio of analyte to IS. Wang et al. [12] showed that the retention time of the [^2^H_5_]-carvedilol-*S* with poorer hydrophobicity was 0.02 min earlier than that of carvedilol-*S*, resulting in greater ionization suppression in carvedilol-*S* and a lower analyte-to-internal standard peak area ratio, and affected the accuracy and precision of quantitative bioanalytical analysis. Therefore, when using deuterated SIL-IS, attention should also be paid to the risk of inaccurate quantification caused by slight retention time differences between SIL-IS and analyte. In this study, although the retention time of SIL-IS GLY-d11 was 0.01 min earlier than that of GLY (Figure 3), GLY-d11 and GLY suffered the same degree of signal suppression, and the ratio of analyte to internal standard did not change, thus ensuring the accuracy of quantification.

### 2.4. Method Validation of GLY

#### 2.4.1. Linearity and LLOQs

To detect the concentration of GLY in the simulated samples, calibration curves containing only GLY were prepared and quantified using the non-isotope internal standard reserpine and the isotope internal standard GLY-d11, respectively. Two methods using reserpine and GLY-d11 as internal standards respectively were validated. Calibration curves were linear over the concentration range of 20–1280 ng/mL for GLY with r^2^ ≥ 0.9855, then analyzed by weighted least-squares (w = 1/x^2^) linear regression analysis. Each method provided the LLOQ of 20 ng/mL for GLY. The intra- and inter-batch precision and accuracy for LLOQ were less than 20% (Table 2).

#### 2.4.2. Accuracy and Precision

Quality control (QC) samples at three GLY concentration levels were used to evaluate the precision and accuracy of both methods. The intra- and inter-batch precision and accuracy of each method are shown in Table 2. These results demonstrated that applied methods were accurate, precise, and reliable for the quantification of GLY.

### 2.5. Pharmacokinetic Study of Simulated Sample

**Figure 5:**
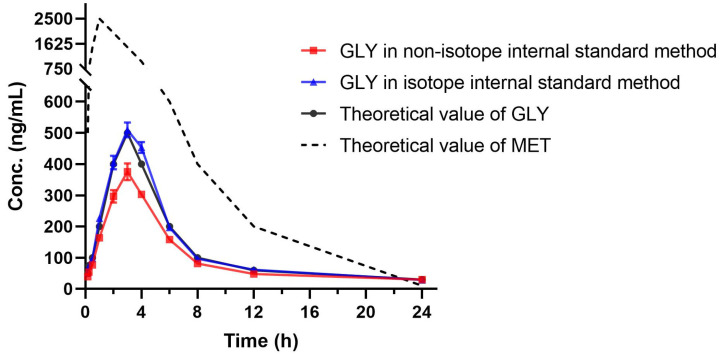
Mean concentration versus time curves obtained under the conditions of non-isotope internal standard and isotope internal standard as well as theoretical curve (*n* = 3). MET: metformin; GLY: glyburide.

The impact of the signal suppression of GLY by MET on pharmacokinetic studies was systematically evaluated. The experiment was designed to explore the signal suppression effect of unpredictable MET in samples on the results of GLY concentration detection when GLY was determined alone. Therefore, the concentration of GLY in the simulated samples containing both GLY and MET was determined using calibration curves containing only GLY. And the simulated samples were analyzed by LC-MS/MS methods using two types of internal standards (reserpine and GLY-d11). Using simulated samples not only conforms to animal welfare and reduces the sacrifice of experimental animals, but also avoids the influence in terms of animals in vivo variation on the experimental results. The mean concentration versus the time curve is shown in Figure 5. The determination concentration and deviation of GLY under the conditions of non-isotope internal standard and isotope internal standard are shown in Table S2 (Supplementary Materials). The results show that when reserpine was used as the internal standard, the deviation of measurement results of GLY in simulation samples was about 25%, which was similar to the signal change rate between five concentration levels of MET and GLY showed in Table 1. From the results in Figure 5, it can also be seen clearly that when reserpine was used as the internal standard, the signal suppression effect of MET on GLY caused the mean concentration versus time curve of GLY to be significantly lower than the theoretical concentration versus time curve. After SIL-IS correction, the concentration deviations of GLY measured at each time point were within 15%, and the mean concentration versus the time curve of GLY coincided with that in theory.

The results indicated that when GLY and MET were co-eluted, the GLY-only calibration standard failed to reflect the suppression between the co-eluting analytes, causing the easily overlooked deviation of detected concentration from the true value. In the clinic, the application of combined drugs is both common and complex. Since the prepared calibration standards and QC plasma samples do not contain co-eluting combination drugs, the suppression between co-elution can hardly be observed during the method validation, let alone its subtle effect on pharmacokinetic study results. If there is a situation similar to the results of this study, the signal suppression effect of co-elution may trick doctors into underestimating the drug concentration in patients, and then affect the reasonable adjustment of drug dose, so the ion suppression between co-elution and their impact on the accuracy and repeatability of quantitation should be carefully analyzed.

In this study, the chromatographic conditions were adjusted to allow the co-elution of MET and GLY, which not only simulated the signal suppression between the potential co-eluting combined drug but also demonstrated the importance of chromatographic separation from the opposite side. Most analysts have realized the necessity of chromatographic separation. However, when the analyst cannot predict the presence of unknown concomitant drugs, there is no guarantee that the validated chromatographic method can avoid the occurrence of signal suppression/enhancement between co-eluting concomitant drugs. Although the SIL-IS has a good correction effect on such signal interference, the authors believe that it is still necessary to understand the information of drug combination as much as possible, and then to analyze the abnormal response and other potential risks caused by ion interference because of the unclear mechanism of ESI. When interference occurs and leads to abnormal results, the investigation of the cause may be complex and disoriented. A simple and rapid method can check for interference: input multiple reaction monitoring (MRM) transitions of possible concomitant drugs present in the sample (which can be obtained from previously published studies) into the mass spectrometry method, and perform sample analysis. If an unknown chromatographic peak overlaps with the chromatographic peak of the analyte, the interference of this substance on the analyte can be further suspected and explored. When such signal suppression is suspected and cannot be effectively separated by chromatography, it is recommended to select standard samples with five concentrations of LLOQ, LQC, MQC, HQC, and ULOQ to explore the mutual interference between co-eluting drugs of different concentrations and further evaluate the impact on quantitative detection.

Generally, during the development and validation of methods, analysts should be aware of possible signal suppression/enhancement between co-eluting drugs to reasonably and effectively solve this problem by chromatographic separation, dilution, SIL-IS correction, and other methods.

## 3. Materials and Methods

### 3.1. Chemicals and Reagents

Metformin Hydrochloride (C_4_H_11_N_5_·HCl, purity 100.0%), glyburide (C_23_H_28_ClN_3_O_5_S, purity > 99.9%), tolbutamide (C_12_H_18_N_2_O_3_S, purity 98%), enalapril maleate (C_20_H_28_N_2_O_5_·C_4_H_4_O_4_, purity 99.8%), indapamide (C_16_H_16_ClN_3_O_3_S, purity 98.2%), nifedipine (C_17_H_18_N_2_O_6_, purity 99.8%), atorvastatin calcium (C_66_H_68_CaF_2_N_4_O_10_·3H_2_O, purity 94.9%), and reserpine (C_33_H_40_N_2_O_9_, purity > 99.9%) were purchased from national institutes for food and drug control (Beijing, China). Glyburide-d11 (C_23_H_17_ClD_11_N_3_O_5_S, purity 98%) was purchased from Toronto Research Chemicals (Toronto, ON, Canada). Methanol (MeOH), acetonitrile (ACN), and methyl tert butyl ether (MTBE) were of HPLC grade and purchased from Fisher Scientific (Pittsburgh, PA, USA). Acetic acid was obtained from Guangzhou chemical reagent factory (Guangzhou, China). Ammonium acetate was purchased from Macklin (Shanghai, China). Ultrapure water was freshly prepared using the Milli-Q Advantage A10 system (Milli-Q Reference, Millipore, Boston, MA, USA). Blank rat plasma samples were harvested from healthy adult rats supplied by the Laboratory Animal Center of Sun Yat-sen University (Guangdong, China).

### 3.2. Chromatographic and Mass Spectrometric Conditions

The HPLC (Thermo Fisher Scientific Inc., Waltham, MA, USA) system consisting of an Ultimate 3000 RSLC system having binary pumps and a Surveyor autosampler (Thermo Fisher Scientific Inc., Waltham, MA, USA) was utilized for this study. Chromatographic separation was performed on a HyPURITY C18 column (150 mm × 2.1 mm, 5 μm; Thermo Scientific, Waltham, MA, USA). Mobile phase A was 2 mM ammonium acetate in water, which was adjusted to pH 5.3 (±0.1) with acetic acid. Mobile phase B was ACN. Under the condition of 65% mobile phase B, GLY and MET were co-eluted and had an identical retention time (2.16 min). The analysis was completed in 4 min at a flow rate of 0.25 mL/min. The injection volume was 2 μL.

A TSQ Quantum Access Max API mass spectrometer (Thermo Fisher Scientific Inc., Waltham, MA, USA) with an ESI source operating in positive ion mode was connected to the LC system for MS detection. The conditions of mass spectrometry were as follows: spray voltage, 4500 V; vaporizer temperature, 350 °C; capillary temperature, 350 °C; sheath gas pressure, 40 psi; aux gas pressure, 20 psi; collision pressure, 1.0 mTorr; the parent ion and daughter ion for each analyte, as well as the related mass spectrum parameters, are shown in Table S3 (Supplementary Materials). Two-stage full-scan mass spectrum of the analytes and internal standards are shown in Figure 1.

### 3.3. Stock Solutions, Calibration Standards and QC Samples Preparation

The stock solution of each analyte and internal standard was prepared in methanol at a concentration of 1 mg/mL, and all were stored at −80 °C. The working solution was prepared by serial dilution with 1:1 methanol-H_2_O (*v/v*). The concentration of the reserpine working solution was 5000 ng/mL, and that of the GLY-d11 working solution was 2400 ng/mL. Calibration standards of GLY were prepared by diluting the working solution with 35:65 mobile phase A-B (*v/v*) at the concentrations of 20, 40, 80, 160, 320, 640, and 1280 (ULOQ) ng/mL. The LLOQ, LQC, MQC, and HQC were prepared with the same procedure at concentrations of 20, 60, 240, and 960 ng/mL for GLY. According to the quantitative range of MET from 50 to 3200 ng/mL, the working solution concentrations of MET at five concentration levels were set and only used for signal suppression analysis between MET and GLY at five concentration levels. The working solutions of MET at five concentration levels of 50 (LLOQ), 150 (LQC), 600 (MQC), 2400 (HQC), and 3200 (ULOQ) ng/mL were prepared under the same procedure. The calibration curve is set according to the concentration range required for pharmacokinetic study [27,28,29]. Finally, the 10 μL internal standard working solution was added into the 100 μL calibration standards or QC samples, vortex mixed and analyzed by LC-MS/MS.

### 3.4. Sample Preparation

#### 3.4.1. Signal Suppression Experiments at Five Concentration Levels

The suppression of analytes during co-eluting was analyzed at the LLOQ, LQC, MQC, HQC, and ULOQ concentration levels of each analyte. The MET work solutions with different concentrations were added into the GLY work solutions, and they were detected simultaneously (obtained Response A of MET or GLY). Meanwhile, the samples containing only MET or GLY at the same concentration were determined individually (obtained Response B of MET or GLY). The ratio of Response A to Response B of MET or GLY was calculated to observe the degree of suppression of MET or GLY at different concentrations and the responses were all corrected by reserpine. Less than 85% of the signal change suggested signal suppression (more than 115% was considered signal enhancement).

#### 3.4.2. Analysis of Plasma Samples

50 μL drug-containing rat plasma and 10 μL internal standard working solution were added to a 1.5 mL centrifuge tube and mixed well. 150 μL ACN and 300 μL MTBE were added for liquid-liquid extraction. Then the mixture was vortex-mixed for 5 min, stood placed for 5 min and centrifuged at 15,000 rpm for 5 min at 4 °C. 360 μL supernatant was transferred to another centrifuge tube and concentrated in a vacuum drying oven, then reconstituted with 35:65 mobile phase A-B (*v/v*). The sample was vortex-mixed for 5 min and then centrifuged at 15,000 rpm for 5 min at 4 °C. Finally, 2 μL supernatant was injected into the LC-MS/MS system.

#### 3.4.3. Analysis of Simulated Pharmacokinetic Samples

Simulated biological samples for pharmacokinetic studies in this study were obtained by diluting the working solution with the mobile phase A-B (35:65, *v/v*). Then, 10 μL internal standard work solution was added into the 100 μL simulated biological samples, vortex mixed and analyzed by LC-MS/MS. Concentration and blood collection time settings for the simulated biological samples were based on previous pharmacokinetic studies of MET and GLY [27,28,29], as shown in Table S2 (Supplementary Materials). The orally administered doses of MET and GLY in this simulated study were 45 and 10 mg/kg, respectively.

### 3.5. Strategies to Solve Signal Suppression of Glyburide by Co-Eluting Metformin

#### 3.5.1. Chromatographic Separation

To separate MET and GLY chromatographically, the mobile phase B (ACN) ratio was increased to 71% (Figure 3B). Considering that combination medication is very common in the clinic, we included possible combination drugs for chromatographic analysis according to the literature [30]. Diabetes is associated with cardiovascular disease, so there may be a potential drug combination of hypoglycemic drugs (tolbutamide), antihypertensive drugs (enalapril, indapamide, nifedipine), and lipid-lowering drugs (atorvastatin).

#### 3.5.2. Sample Dilution

To test whether dilution could solve the signal suppression of GLY by MET, the LLOQ, LQC, MQC, HQC, and ULOQ samples of MET and GLY were diluted 10-or 20-fold, and other experimental procedures were the same as 3.4.1.

#### 3.5.3. Correction of Stable-Isotope-Labeled Internal Standard

To test whether the SIL-IS could correct the signal suppression of GLY by MET, 10 μL GLY-d11 working solution was added into the 100 μL sample and the final concentrations of MET and GLY remained the same as in Section 3.4.1. In the analysis of simulated pharmacokinetic samples, GLY-d11 was used as SIL-IS, and other experimental procedures were the same as in Section 3.4.3. The degree of signal suppression of GLY or GLY-d11 was calculated, respectively, as calculated in Section 3.4.1. Then, the ratio of the two signal suppression rates was further calculated. When the ratio is between 85% and 115%, it means that GLY-d11 as the internal standard can correct the signal of GLY suppressed by MET, otherwise it cannot correct the signal suppression.

### 3.6. Method Validation

The validation of the assay method for GLY detection alone was assessed in terms of linearity, precision, and accuracy according to the bioanalytical method validation guidance of US Food and Drug Administration [15]. The matrix effect and recovery of MET and GLY were also determined according to the guidance.

### 3.7. Statistical Analysis and Software

The raw data was sorted using Microsoft Excel. Statistical analysis used Statistical Package for the Social Sciences (SPSS) (version 20.0, SPSS Inc, Chicago, IL, USA), and plotting used GraphPad Prism 8 (GraphPad Software Inc., San Diego, CA, USA). The Xcalibur software was used to establish the calibration curves fitted with weighted (1/x^2^) and to calculate the accuracy and precision of the QC samples (*n* = 6).

## 4. Conclusions

In this study, a co-eluting model was established to explore the impact of signal suppression caused by co-eluting combined drugs on biological sample detection and pharmacokinetic analysis. The result concerning the signal suppression of MET and GLY at five concentrations indicated that the signal of GLY can be significantly suppressed by the high concentration of MET. Some strategies have been employed to solve the signal suppression of GLY by MET. Chromatographic separation of co-eluting combined drugs is effective, but in the presence of multiple combined drugs, new co-eluting drugs may arise after chromatographic separation to produce signal suppression. In this study, the sample dilution not only failed to completely solve the signal suppression of MET on GLY but also affected the sensitivity and precision of the detection. The degree of signal suppression of MET on GLY and GLY-d11 was similar, so the correction strategy of SIL-IS was feasible in this paper. Further pharmacokinetic analyses using simulated samples were therefore performed. Without the correction of SIL-IS, the deviation of measurement results of GLY in simulated samples was about 25%. This indicates that signal suppression between co-eluting concomitant medications can affect pharmacokinetic analysis and may also affect a series of biological analyses based on LC-MS/MS, including therapeutic drug monitoring, bioequivalence study, and so on. In conclusion, concomitant drugs are common in clinical practice, and signal suppression between concomitant drugs in LC-MS/MS analysis requires more attention to avoid its potential risk to quantitative accuracy.

## Figures and Tables

**Table 1:** Co-analytes signal interference at five concentration levels in the standard solution sample and plasma sample (*n* = 3). Standard solution sample: theoretical concentration, diluted 10 times and 20 times; Plasma sample: sample after pretreatment. It is marked orange when the signal is suppressed below 85%. Conc.: concentration; LLOQ: lower limit of quantification; LQC: low-quality control; MQC: medium-quality control; HQC: high-quality control; ULOQ: upper limit of quantitation. MET: metformin; GLY: glyburide.

		MET Conc. (ng/mL)									
		LLOQ (50)	LQC (150)	MQC (600)	HQC (2400)	ULOQ (3200)	LLOQ (50)	LQC (150)	MQC (600)	HQC (2400)	ULOQ (3200)
	GLY Conc. (ng/mL)	Degree of Signal Suppression of GLY by MET (%)					Degree of Signal Suppression of MET by GLY (%)				
Nominal concentration	LLOQ (20)	87	88	74	72	66	93	99	92	111	113
	LQC (60)	89	86	74	73	72	94	90	91	101	100
	MQC (240)	93	91	80	77	71	106	105	103	95	100
	HQC (960)	87	90	81	71	69	106	95	108	88	110
	ULOQ (1280)	92	90	79	74	73	113	98	106	89	111
10-fold dilution for MET and GLY	LLOQ (20)	106	91	101	76	73	89	111	107	114	85
	LQC (60)	102	106	103	82	81	109	105	91	115	87
	MQC (240)	105	93	105	88	89	90	103	97	102	90
	HQC (960)	96	95	96	92	89	95	111	99	95	94
	ULOQ (1280)	89	92	92	88	93	93	110	106	96	104
20-fold dilution for MET and GLY	LLOQ (20)	107	109	91	71	75	91	110	95	93	100
	LQC (60)	114	101	102	82	92	110	95	92	86	105
	MQC (240)	102	97	99	96	87	99	107	89	93	99
	HQC (960)	87	101	108	92	91	106	114	98	92	94
	ULOQ (1280)	89	106	87	90	88	99	113	89	86	94
Plasma samples for MET and GLY	LLOQ (20)	88	82	76	79	77	94	100	96	97	104
	LQC (60)	92	82	85	84	81	92	107	107	102	106
	MQC (240)	90	87	83	80	81	104	106	88	91	110
	HQC (960)	94	90	93	83	85	100	96	98	94	106
	ULOQ (1280)	103	97	93	95	88	91	97	91	91	101

**Table 2:** Precision and accuracy of LC-MS/MS to determine glyburide (GLY) under the conditions of non-isotope internal standard and isotope internal standard (mean ± SD, *n* = 6). SD: standard deviation; CV: coefficient of variation.

		Intar Run (*n* = 6)			Inter Run (*n* = 18)		
	Nominal Conc. (ng/mL)	Calculated Conc. (ng/mL)	Accuracy (%)	CV (%)	Calculated Conc. (ng/mL)	Accuracy (%)	CV (%)
Non-isotope internal standard method	20	20.8 ± 2.6	3.8	12.7	20.2 ± 0.9	0.8	4.3
	60	63.5 ± 7.5	5.9	11.7	59.4 ± 4.7	−1.1	7.8
	240	234.4 ± 19.3	−2.3	8.3	235.6 ± 18.5	−1.8	7.9
	960	904.0 ± 75.8	−5.8	8.4	981.3 ± 86.1	2.2	8.8
Isotope internal standard method	20	21.8 ± 1.4	9.0	6.3	20.4 ± 1.2	2.0	6.0
	60	61.3 ± 1.8	2.2	3.0	60.4 ± 1.0	0.7	1.7
	240	244.7 ± 10.3	2.0	4.2	241.7 ± 5.1	0.7	2.1
	960	932.5 ± 35.5	−2.9	3.8	943.0 ± 9.3	−1.8	1.0

## Data Availability

Not applicable.

## Supplementary Materials

The following supporting information can be downloaded at: https://www.mdpi.com/article/10.3390/molecules28020746/s1, Figure S1: Effect of protein precipitating reagent and extraction solvent volume on recovery and matrix effect (*n* = 3). (A) MET recovery, (B) GLY recovery, (C) MET matrix effect, (D) GLY matrix effect; Table S1: Recovery and matrix effect for the determination of MET and GLY in rat plasma. (mean ± SD, *n* = 3); Table S2: Blood collection time and drug concentration of simulated biological samples, as well as the determination concentration and deviation of GLY under the conditions of non-isotope internal standard and isotope internal standard (*n* = 3); Table S3: The parent ion and daughter ion for each analyte as well as the related mass spectrum parameters.

## Sample Availability

Samples of the compounds are not available from the authors.
