# Supplementary material for: Signal Suppression in LC-ESI-MS/MS from Concomitant Medications and Its Impact on Quantitative Studies: An Example Using Metformin and Glyburide
Source: Molecules. 2023 Jan 11;28(2):746. doi: 10.3390/molecules28020746 (PMC9862991; doi:10.3390/molecules28020746)
Supplement: Supplementary file 1 [file molecules-28-00746-s001.zip › molecules-2120752-supplementary.pdf]

Supplementary Materials

Signal Suppression in LC-ESI-MS/MS from Concomitant Medications and Its Impact on Quantitative Studies: An Example Using Metformin and Glyburide

Jingyu Liu [1,†], Fulin Jiang [1,2,†], Zihan Lu [1], Chang Zhang [1], Peiqing Liu [2], Min Huang [1,*] and Guoping Zhong [1,*]

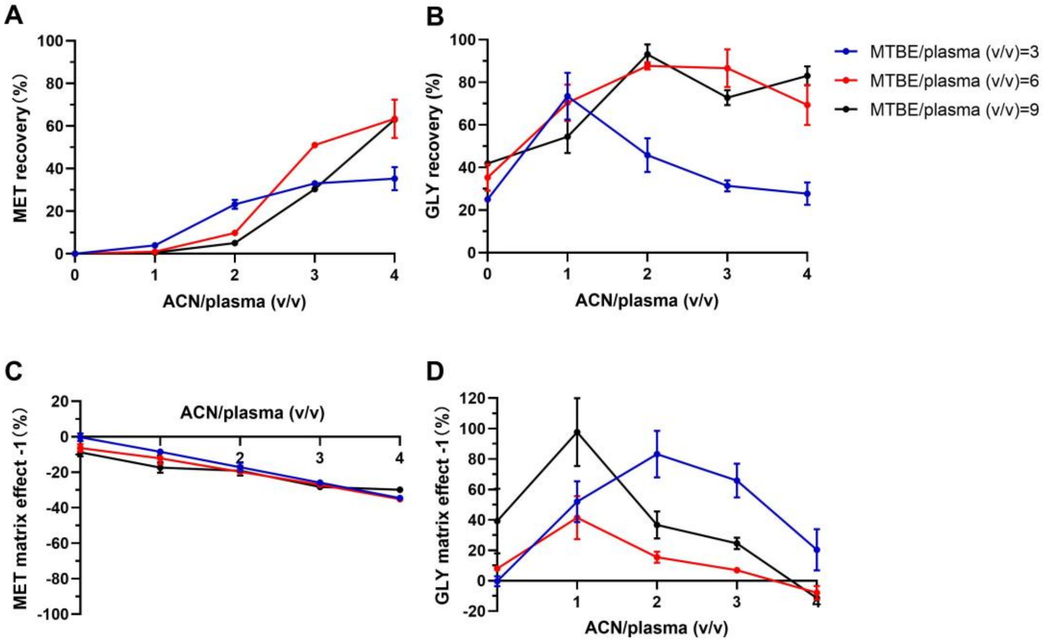

Figure S1. Effect of protein precipitating reagent and extraction solvent volume on recovery and matrix effect (*n* = 3). (**A**) MET recovery, (**B**) GLY recovery, (**C**) MET matrix effect, (**D**) GLY matrix effect. MTBE: Methyl tert butyl ether; ACN: acetonitrile.−

Table S1. Recovery and matrix effect for the determination of MET and GLY in rat plasma. (mean ± SD, *n* = 3).

		**Recovery (%)**		**Matrix effect (%)**	
		Mean ± SD	RSD (%)	Mean ± SD	RSD (%)
MET	LQC	21.5 ± 1.7	8.1	97.1 ± 9.0	9.3
	MQC	24.4 ± 1.2	5.1	94.7 ± 4.7	5.0
	HQC	28.2 ± 1.0	3.7	92.4 ± 4.7	5.1
GLY	LQC	60.9 ± 7.2	11.9	91.1 ± 12.1	13.3
	MQC	66.9 ± 5.8	8.6	109.2 ± 4.2	3.9
	HQC	58.1 ± 1.9	3.2	110.9 ± 4.2	3.8

Table S2. Blood collection time and drug concentration of simulated biological samples, as well as the determination concentration and deviation of GLY under the conditions of non-isotope internal standard and isotope internal standard (*n* = 3). RE: relative error.

**Time (h)**	**Theoretical Conc. (ng/mL)**		**Non-isotope internal standard method**		**Isotope internal standard method**	
	**MET**	**GLY**	**Calculated Conc. of GLY (ng/mL)**	**RE (%)**	**Calculated Conc. of GLY (ng/mL)**	**RE (%)**
0.17	500	50	39.2 ± 3.3	−21.6	51.0 ± 1.7	2.0
0.25	750	75	54.1 ± 1.8	−27.8	73.1 ± 2.9	−2.5
0.5	1500	100	77.0 ± 4.1	−23.0	97.3 ± 7.3	−2.7
1	2500	200	163.6 ± 2.7	−18.2	226.3 ± 9.1	13.1
2	2000	400	296.6 ± 19.8	−25.9	405.0 ± 21.7	1.2
3	1500	500	375.8 ± 26.6	−24.9	510.0 ± 23.6	2.0
4	1000	400	303.3 ± 0.6	−24.2	453.5 ± 17.4	13.4
6	600	200	−158.0 ± 1.1	−21.0	196.8 ± 7.2	−1.6
8	400	100	81.5 ± 1.8	−18.6	96.7 ± 4.4	−3.3
12	200	60	48.2 ± 1.5	−19.6	61.9 ± 3.5	3.2
24	10	30	30.3 ± 0.8	1.0	28.9 ± 2.1	−3.6

Table S3. The parent ion and daughter ion for each analyte as well as the related mass spectrum parameters.

**Analytes**	**Parent ion (m/z)**	**Daughter ion (m/z)**	**Collision energy (V)**
Metformin	130.1	60.4	13
Glyburide	494.3	369.3	13
Tolbutamide	271.3	91.3	31
Enalapril	377.4	234.2	19
Indapamide	366.3	132.3	14
Nifedipine	347.4	254.2	18
Atorvastatin	559.5	440.5	20
Reserpine	609.5	397.1	32
Glyburide-d11	505.4	369.3	13
